# 肿瘤骨转移治疗的系统评价

**DOI:** 10.3779/j.issn.1009-3419.2010.05.28

**Published:** 2010-05-20

**Authors:** 尧尧 任, 力 马, 金徽 田, 琳琳 张, 克虎 杨

**Affiliations:** 1 730000 兰州，兰州大学循证医学中心，兰州大学基础医学院 Evidence-based Medicine Center of Lanzhou University, Basic Medical of Lanzhou University School, Lanzhou 730000, China; 2 300052 天津，天津医科大学总医院肿瘤内科 Department of Medical Oncology, Tianjin Tocacic Cancer Center, Tianjin Medical University General Hospital, Tianjin 300052, China

**Keywords:** 放疗, 双膦酸盐, 骨转移, 系统评价, Radiotherapy, Bisphosphonates, Bone metastasis, Systematic review

## Abstract

**背景与目的:**

骨转移是晚期恶性肿瘤常见并发症之一。本研究系统评价放疗联合静脉双膦酸盐与单纯放疗治疗肿瘤骨转移的临床疗效和安全性。

**方法:**

计算机检索Cochrane图书馆、PubMed、EMBASE、CBM、CNKI和VIP等数据库，手工检索相关中文期刊，查阅纳入文献的参考文献，按照Cochrane系统评价方法评价纳入研究的质量，采用RevMan 5.0软件进行统计分析。

**结果:**

共纳入22项研究，1 585例病例。*meta*分析结果显示：放疗联合静脉双膦酸盐疗法在疼痛控制总有效率（RR=1.21, 95%CI: 1.13-1.30, *P* < 0.001）、平均缓解时间（WMD=16.00, 95%CI: 10.12-21.88, *P* < 0.001）、病灶控制率（RR=1.69, 95%CI: 1.43-1.99, *P* < 0.001）和生活质量改善总有效率（RR=1.25, 95%CI: 1.08-1.45, *P*=0.003）方面优于单纯放疗。在不良反应方面，除放疗联合静脉双膦酸盐疗法发热高于单纯放疗外（RR=5.61, 95%CI: 3.11-10.13, *P* < 0.001），在恶心呕吐发生率（RR=1.15, 95%CI: 0.62-2.13, *P*=0.65）和骨髓抑制发生率（RR=1.23, 95%CI: 0.75-2.02, *P*=0.40）方面差异无统计学意义。

**结论:**

放疗联合静脉双膦酸盐可快速、有效并长效控制骨转移性疼痛，有效提高肿瘤患者的活动能力及生活质量。

## Abstract (en)

**Background and objective:**

Skeletal metastase is one of the most common complications related to advanced cancer. The aim of this study is to analyze the effectiveness and safety of radiotherapy plus intravenous bisphosphonates versus radiotherapy alone for treating bone metastasis.

**Methods:**

We searched the Cochrane Library, PubMed, EMBASE, CBM, CNKI and VIP, as well as the reference lists of reports and reviews. The quality of included trials was evaluated by the Cochrane Handbook. Data were extracted and evaluated by two reviewers independently. The Cochrane Collaboration's Rev-Man 5.0 was used for data analysis.

**Results:**

Twenty-two trials involving 1 585 patients were included. Compared with radiotherapy alone, radiotherapy plus intravenous bisphosphonates was more effective in total effective rate of pain relive (RR=1.21, 95%CI: 1.13-1.30, *P* < 0.001), average abated time (WMD=16.00, 95%CI: 10.12-21.88, *P* < 0.001), and quality of life (RR=1.25, 95%CI: 1.08-1.45, *P*=0.003, with significant differences. Side effects have no significant differences between the two groups except fever (RR=5.61, 95%CI: 3.11-10.13, *P* < 0.001).

**Conclusion:**

Current evidence supports more effective of radiotherapy plus intravenous bisphosphonates for bone metastases. The combine treatment is safe and effective.

骨转移是晚期恶性肿瘤患者常见并发症之一，特别是乳腺癌、前列腺癌、肺癌、甲状腺癌和肾癌等最易出现骨转移^[1]^。60%-70%的乳腺癌患者会发生一处或多处骨转移^[2]^；前列腺癌患者的骨转移发生率高达65%-75%^[3]^。骨转移产生明显的临床症状及并发症称为骨相关事件^[4]^，包括骨痛、病理性骨折、脊髓压迫和高钙血症，使患者食欲减退，睡眠质量降低，活动障碍，生活质量严重下降。45%-75%的骨转移患者出现疼痛和继发的活动障碍，增加了癌症患者的痛苦^[5]^。

虽然放疗可缓解骨痛、减少病理性骨折危险，但单纯放疗对广泛骨转移的治疗应用受限，且某些部位的转移灶如脊柱转移灶受放射剂量限制，故临床希望采用放疗联合双膦酸盐治疗的方式，以期能快速且持久止痛，减少骨相关事件发生，提高生活质量。有研究^[6-10]^显示联合治疗在疼痛控制方面优于单纯放疗，但也有研究显示两种疗法在疼痛控制方面无统计学差异。本研究拟采用系统评价的方法客观评价放疗联合静脉双膦酸盐治疗骨转移的疗效。

## 1 材料与方法

### 1.1 纳入标准

#### 1.1.1 研究类型

随机对照试验，无论是否采用盲法。

#### 1.1.2 研究对象

有原发灶且经病理或细胞学证实为恶性肿瘤的患者，经X线、ECT、MR或CT影像学证实有单发或多发骨转移灶。

#### 1.1.3 干预措施

外照射放疗联合静脉双膦酸盐vs外照射放疗。

#### 1.1.4 观察指标

止痛总有效率、疼痛完全缓解率、起效时间、平均缓解时间、病灶控制率、活动能力改善及生活质量提高程度、副作用。

### 1.2 检索策略

以“（放疗OR外照射）AND（双膦酸盐OR二膦酸盐OR帕咪膦酸二钠OR唑来膦酸OR伊班膦酸钠）”为检索词，并根据具体数据库调整，所有检索采用主题词[MEDLINE (MeSH), EMBASE (EMTREE)]与自由词相结合的方式，所有检索策略通过多次预检索后确定。

### 1.3 文献筛选和资料提取

2位研究者独立阅读所获文献题目和摘要，在排除明显不符合纳入标准的试验后，对可能符合纳入标准的试验阅读全文，以确定其是否纳入。2位研究者交叉核对纳入试验的结果，对有分歧而难以确定的试验通过讨论或由第3位研究者决定其是否纳入。缺乏的资料通过电话或信件与作者进行联系予以补充。

从文献中提取的信息包括：①一般资料：题目、作者、发表日期和文献来源；②研究特征：研究对象的一般情况、患者的基线情况、干预措施；③结局指标：止痛总有效率、中位缓解时间、病灶控制率、生活质量提高程度，治疗引起的并发症包括发热、恶心呕吐及骨髓抑制。

### 1.4 质量评价

纳入文献的方法学质量依据Cochrane评价手册5.0随机对照试验质量的4条质量评价标准进行评价。

### 1.5 统计分析

采用Cochrane协作网提供的RevMan 5.0统计软件进行*meta*分析。计数资料采用相对危险度（RR）为疗效分析统计量；剂量资料采用加权均数差（WMD）或标准化均数差（SMD），并计算各效应量的95%可信区间（95%CI）。各纳入研究结果间的异质性采用χ^2^检验，若纳入研究具有足够一致性（*P* > 0.1和*I*^2^ < 50%）时，采用固定效应模型进行分析，若纳入研究存在异质性时，分析其异质性来源，对可能导致异质性的因素进行亚组分析。若各研究间存在统计学异质性而无临床异质性或差异无临床意义时，采用随机效应模型分析。以*P* < 0.05为差异有统计学意义。

## 2 结果

### 2.1 纳入研究一般情况

初检相关文献345篇，阅读标题和摘要，排除重复64篇、综述5篇、病例报告1篇、无具体实质性内容209篇，非随机15篇。剩下51篇查找原文进一步确定，其中25篇未达到纳入标准被排除，4篇非随机，最终22篇^[6-27]^符合纳入标准，共有患者1 585例。

### 2.2 方法学质量评价

#### 2.2.1 随机方法

22项研究^[6-27]^均提及随机，但未描述具体随机方法。

#### 2.2.2 分配隐藏

2项研究^[9, 18]^采用信封，其余均未提及。

#### 2.2.3 盲法

1项研究^[13]^实施双盲，但未详细描述，其余均未提及。

#### 2.2.4 失访

22项研究^[6-27]^均无失访。

### 2.3 *meta*分析结果

#### 2.3.1 有效性

##### 2.3.1.1 止痛总有效率

22项研究^[6-27]^报道了止痛总有效率，各研究间有统计学异质性（*P* < 0.001, *I*^2^=68%），采用随机效应模型分析（图 1）。结果显示放疗联合静脉双膦酸盐控制疼痛的总有效率优于单纯放疗组（RR=1.21, 95%CI: 1.13-1.30, *P* < 0.001）。因病例数差异剔除3项研究^[9, 13, 19]^后再作*meta*分析，结果无明显变化（RR=1.14, 95%CI: 1.09-1.20），提示*meta*分析结果稳定（图 2）。

**1:**
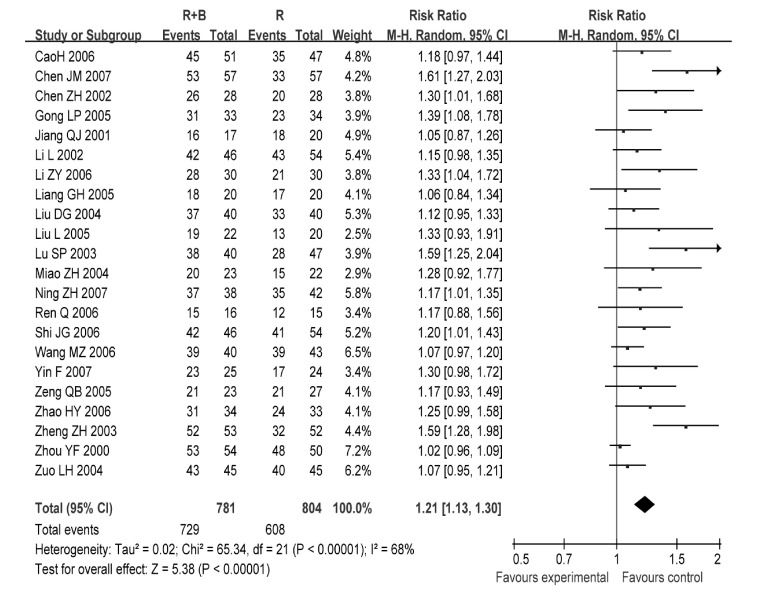
放疗联合双膦酸盐与放疗控制疼痛的总有效率比较 Comparison radiotherapy alone *vs* radiotherapy plus bisphosphonates, outcome risk ratio for overall effective

**2:**
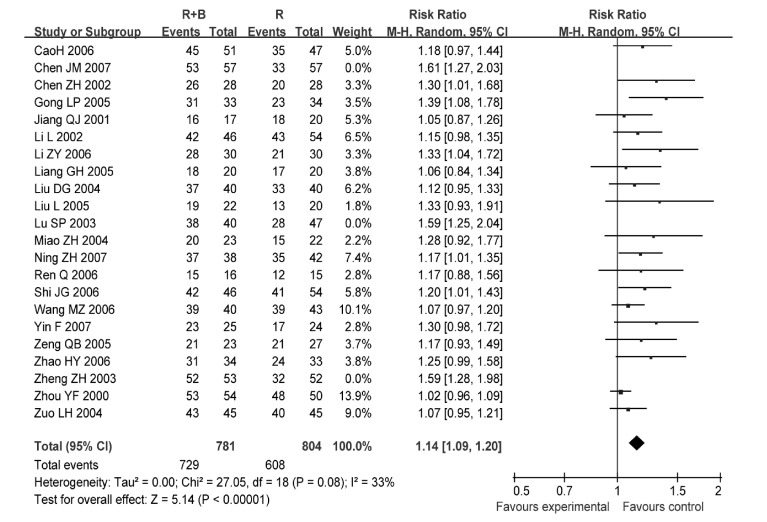
敏感性分析后的放疗联合双膦酸盐对比放疗控制疼痛的总有效率 Comparison radiotherapy alone *vs* radiotherapy plus bisphosphonates, outcome risk ratio for overall effective, after sensitivity analysis

##### 2.3.1.2 平均缓解时间

2项研究报告了平均缓解时间^[7, 25]^，研究间有统计学异质性（*P*=0.04, *I*^2^=77%），采用随机效应模型进行分析，结果显示放疗联合双膦酸盐可以明显延长疼痛平均缓解时间（WMD=16.00, 95%CI: 10.12-21.88, *P* < 0.001）（图 3）。

**3:**
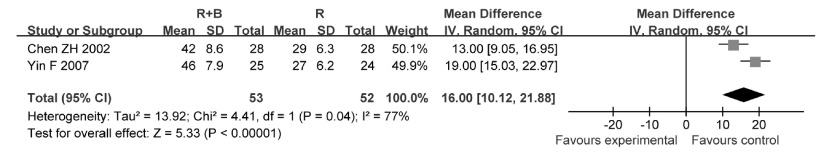
放疗联合双膦酸盐止痛平均缓解时间 Comparison radiotherapy alone *vs* radiotherapy plus bisphosphonates, outcome mean difference for abated time

##### 2.3.1.3 病灶控制率

8项研究^[9, 15, 18, 21, 23-25, 27]^报告了病灶控制率，各研究间无统计学异质性（*P*=0.47, *I*^2^=0%），采用固定效应模型进行分析，结果显示联合使用双膦酸盐组的病灶控制率高于单纯放疗组（RR=1.69, 95%CI: 1.43-1.99, *P* < lt; 0.001）（图 4）。

**4:**
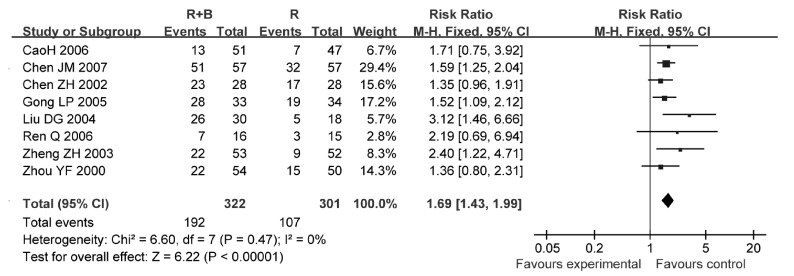
放疗联合双膦酸盐与放疗的病灶控制率比较 Comparison radiotherapy alone *vs* radiotherapy plus bisphosphonates, outcome risk ratio for lesion control rate

##### 2.3.1.4 生活质量改善总有效率

4项研究^[6, 12, 21, 24]^报道了生活治疗提高总有效率，各研究间无统计学异质性（*P*=0.17, *I*^2^=41%），采用固定效应模型进行分析，结果显示联合使用双膦酸盐组患者生活质量提高总有效率高于单纯放疗组（RR=1.25, 95%CI: 1.08-1.45, *P*=0.003）（图 5）。

**5:**
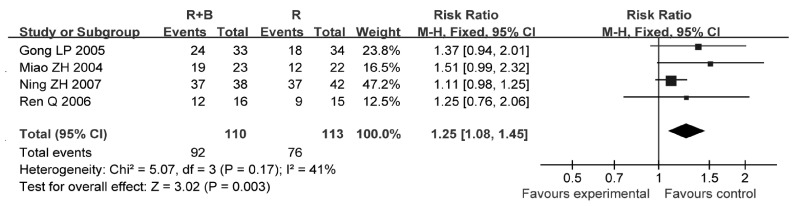
放疗联合双膦酸盐与放疗的生活质量改善情况比较 Comparison radiotherapy alone *vs* radiotherapy plus bisphosphonates, outcome risk ratio for improved quality of life

#### 2.3.2 安全性

##### 2.3.2.1 发热

12项研究^[6, 7, 8, 11, 15-17, 19-21, 24, 25]^报告了发热不良反应，各研究间无统计学异质性（*P*=0.39, *I*^2^=5%），采用固定效应模型进行分析，结果显示联合使用双膦酸盐组患者发热发生率率高于单纯放疗组，差异有统计学意义（RR=5.61, 95%CI: 3.11-10.13, *P* < 0.001）（图 6）。

**6:**
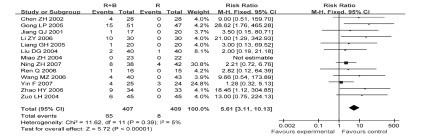
放疗联合双膦酸盐与放疗的发热发生率比较 Comparison radiotherapy alone *vs* radiotherapy plus bisphosphonates, outcome risk ratio for fever

##### 2.3.2.2 恶心呕吐发生率

8项研究^[6, 7, 8, 12, 17, 21, 23, 25]^报告了恶心呕吐发生率，各研究间无统计学异质性（*P*=0.38, *I*^2^=6%），采用固定效应模型分析，结果显示两组患者恶心呕吐发生率差异无统计学意义（RR=1.15, 95%CI: 0.62-2.13, *P*=0.65）（图 7）。

**7:**
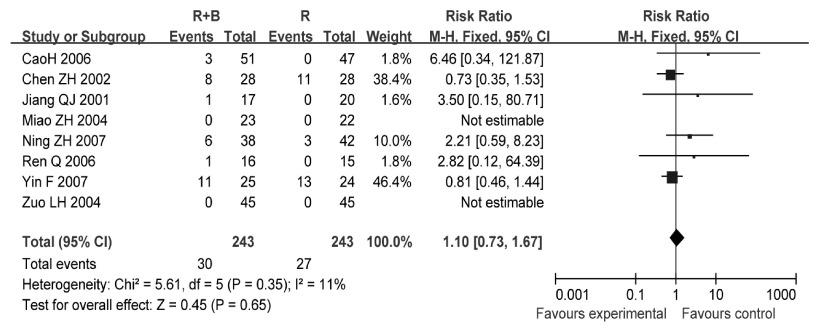
放疗联合双膦酸盐与放疗的恶心呕吐发生率比较 Comparison radiotherapy alone *vs* radiotherapy plus bisphosphonates, Outcome Risk Ratio for nausea and vommiting

##### 2.3.2.3 骨髓抑制发生率

10项研究^[6, 7, 11, 12, 15-17, 20, 21, 25]^报道了骨髓抑制发生情况，各研究间无统计学异质性（*P*=0.97, *I*^2^=0%），采用固定效应模型分析，结果显示两组患者骨髓抑制发生率差异无统计学意义（RR=1.23, 95%CI: 0.75-2.02, *P*=0.40）（图 8）。

**8:**
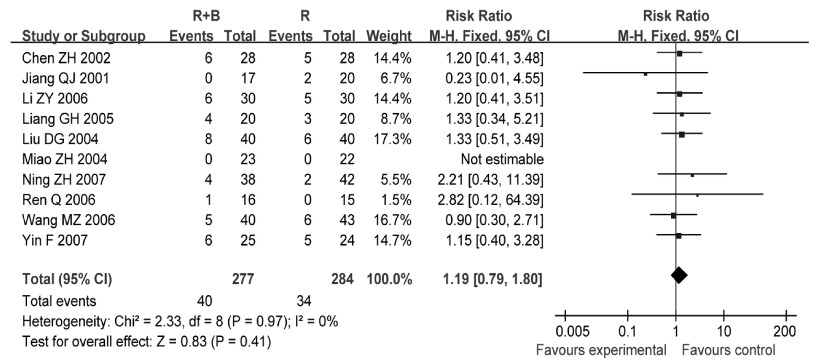
放疗联合双膦酸盐与放疗的骨髓抑制发生率比较 Comparison radiotherapy alone *vs* radiotherapy plus bisphosphonates, Outcome Risk Ratio for myelosuppression

## 3 讨论

*meta*分析结果显示放疗联合静脉双膦酸盐治疗方法在疼痛控制总有效率、疼痛完全缓解率、起效时间、中位缓解时间、病灶控制率、活动能力改善及生活质量改善总有效率方面优于单纯放疗。在疼痛总有效率的分析中，各研究间存在较大异质性，分析原因可能为其中3项研究^[9, 13, 27]^所使用的止痛疗效评定指标与其它研究不一致。敏感性分析后结果稳定，提示联合治疗可以迅速、有效并长效控制疼痛，提高患者生活质量。2个报告了平均缓解时间的研究效应量存在异质性（*I*^2^=77%），分析原因是因为2项研究使用的双膦酸盐药物不同。尹锋等^[7]^研究使用伊班膦酸钠，陈梓宏等^[25]^研究使用帕咪膦酸二钠，二者间存在临床异质性。在治疗的安全性方面，联合使用双膦酸盐组的发热发生率高于单纯放疗组，当前证据^[28]^表明静脉双膦酸盐注射后会有一过性可控制的流感样症状，治疗相关性发热均为轻-中度发热，对症处理后缓解。在消化道毒性、骨髓抑制方面两组均无差异，因此，放疗联合静脉双膦酸盐不增加消化道毒性及骨髓抑制。

本系统评价所纳入22项研究均有明确的诊断标准；其中11项研究描述了基线情况，两组间基线情况一致使之具有可比性。但研究质量相对较低。纳入文献均提及随机，但未明确描述随机方法；只有2项研究采用信封实施分配隐藏^[9, 18]^，其它均未提及，随机方法不充分会造成选择性偏倚，而分配方案隐藏不完善可能导致治疗结果被夸大。1项研究采用双盲^[13]^，但未描述盲谁、如何盲，这将会导致实施偏倚存在。对于计量资料的统计分析，部分研究未提供两组治疗前后的均数和标准差，故无法进行*meta*分析。本系统评价所采用资料均为已发表的文献，缺乏如专题报道、未发表的资料、政府报告或非传统文献来源的证据。

本系统评价纳入文献均为中文文献，国外尚未见同类研究，限制了研究结论的普适性。分析原因，根据WHO三阶梯止痛原则，对于癌性疼痛首选治疗为镇痛药物，通过剂量滴定快速有效止痛，属于对症治疗，因此在国外，首选镇痛药物止痛治疗。而在我国由于患者对阿片类镇痛药物的恐惧、对镇痛药物治疗的依从性较差，放疗及静脉双膦酸盐成为临床应用最多的治疗方式。放疗除缓解局部骨痛外，治疗的机制在于其直接的抗肿瘤效应和抑制骨转移相关的破骨细胞活性^[29]^。双膦酸盐能与骨基质中的无机物结合，且优先结合骨重建活跃的区域，结合后，直接影响成熟破骨细胞的再吸收活性，能够抵抗破骨细胞的溶解作用，延缓转移的发展，因此除控制疼痛外，放疗和双膦酸盐更可减少病理性骨折等骨相关事件，从而进一步提高癌症患者的生活质量。值得注意的是，双膦酸盐的治疗优势需6个月以上才能明显显现^[30]^。所以需要长期随访观察，本系统研究所纳文献，均无6个月以上的长期随访报告，故双膦酸盐对远期指标的影响未得到充分体现。
